# Concordance between biomarkers in cerebrospinal fluid (CSF), brain positron emission tomography with 18‐fluorodeoxyglucose (18F‐FDG‐PET) and clinical diagnosis in patients with cognitive impairment

**DOI:** 10.1002/alz.088290

**Published:** 2025-01-09

**Authors:** Alberto Jose Mimenza, Karen Leon del Ángel, Rodrigo Hernández Martínez, Sara Gloria Aguilar‐Navarro

**Affiliations:** ^1^ Instituto Nacional de Ciencias Médicas y Nutrición Salvador Zubirán, México, DF Mexico; ^2^ Instituto Nacional de Ciencias Médicas y Nutrición Salvador Zubiran, Mexico, EM Mexico; ^3^ Instituto Nacional de Ciencias Médicas y Nutrición Salvador Zubirán, Mexico, EM Mexico

## Abstract

**Background:**

The diagnosis of neurodegenerative diseases such as dementia due to Alzheimer's Disease (AD) and other dementias continues to be a challenge. The use of complementary studies in diagnosis is recommended in the main clinical guidelines for this pathology. Biomarkers in CSF (Aβ42, tTau and pTau) and by brain [18F]FDG‐PET are useful for diagnosis, since they increase its certainty. Objective: Establish diagnostic agreement between biomarkers in CSF, PET and clinical evaluation.

**Method:**

Cohort, retrospective and comparative study. Patients with biomarkers were included for the diagnostic approach of cognitive impairment due to any cause in the INCMNSZ from July 2018 to September 2023. An analysis of concordance was carried out with the Kappa coefficient between the result of the biomarkers and the clinical assessment.

**Result:**

77 patients were evaluated, with an average age of 71 years (SD ± 10.19) and 53.2% were men; 27.3% with a diagnosis of mild cognitive impairment (MCI) and 72.7% with dementia. The agreement between the initial diagnosis and the hypometabolism patterns by PET was moderate (kappa 0.461, p=0.011). The overall agreement between the biomarkers to differentiate between AD and other dementias was 88% (kappa 0.694, p=0.002). The change in diagnosis after taking the biomarkers occurred in 46% of the patients.

**Conclusion:**

This study demonstrates that the agreement between the use of CSF and PET biomarkers to differentiate between AD and other dementias is good. There was disagreement in 12% of the patients, which suggests that in cases with diagnostic doubt it is advisable to perform both biomarkers.

